# Large-scale functional overlap between dorsal and ventral object-responsive networks

**DOI:** 10.21203/rs.3.rs-9180836/v1

**Published:** 2026-04-16

**Authors:** Claire Simmons, Marlene Behrmann, Vladislav Ayzenberg

**Affiliations:** 1 Department of Psychology and Neuroscience Institute, Carnegie Mellon University, Pittsburgh, PA, USA; 2 School of Medicine, University of Pittsburgh, Pittsburgh, PA, USA; 3 Department of Ophthalmology, University of Pittsburgh, PA, USA; 4 Department of Psychology and Neuroscience, Temple University

**Keywords:** Higher-order visual areas, Object recognition, Dorsal parietal cortex, Ventral temporal cortex, Functional connectivity, RRID:_023356

## Abstract

The processes of recognizing objects and acting upon or with them have traditionally been attributed to computations associated with ventral and dorsal visual pathways, respectively. Accumulating evidence indicates that specific regions of the two pathways interact but the extent and strength of the whole-brain network connectivity remain to be evaluated. Here, in two experiments, we characterized the whole-brain network connections of object-selective seeds in each pathway using multiple analytic approaches with functional MRI data acquired while participants viewed objects. The results revealed substantial spatial overlap in connectivity in the networks generated from a dorsal and a ventral seed — with the greater similarity of these object networks within an individual than the similarity of each network separately across individuals. This overlap persisted for both tools and non-tools even after controlling for the shared variance between pathways. Notably, the dorsal pathway showed overall stronger and more widespread connectivity than the ventral pathway and was a stronger source of effective connectivity across the brain. Together, these findings reveal that dorsal and ventral pathways form highly overlapping and distributed networks in the service of object perception.

## INTRODUCTION

With merely a glance, humans can identify an object, appreciate the actions it affords and interact with it. These competencies are not limited to objects encountered frequently, such as identifying a key to open the front door or using a comb to brush one’s hair, but are also generalizable to new contexts, such as using a rolling pin as an impromptu hammer. Despite their apparent entanglement in everyday life, the processes of object recognition and action have traditionally been attributed to the functions of two distinct processing pathways: a dorsal parietal “where/how” pathway subserving spatial and action-based representation of objects and a ventral temporal “what” pathway mediating object recognition (Goodale & Milner, 1992; Mishkin & Ungerleider, 1982). However, since this initial formulation, accumulating evidence suggests that dorsal and ventral pathways are less separable than previously assumed and, instead, each shares computational functions typically ascribed to just one of the pathways.

Extensive research has shown, for example, that dorsal cortex is also involved in object recognition (Freud et al., 2016; Xu & Vaziri-Pashkam, 2019), contributing to it by computing important shape properties such as global structure (Ayzenberg & Behrmann, 2022; Georgieva et al., 2008; Sablé-Meyer et al., 2026) and three-dimensional form (Freud et al., 2015; Georgieva et al., 2008; Orban, 2011). Consistent with this role of dorsal cortex in representing shape information, damage to parietal cortex impairs processes critical for object recognition, such as the ability to match shapes (Riddoch et al., 2008) and to integrate local elements into a global form (Romei et al., 2011), and transient inactivation of the caudal intraparietal area in non-human primates impairs their perception of an object’s 3D structure (Van Dromme et al., 2016).

While there is a clear consensus that the ventral visual pathway plays the pre-eminent role in subserving object recognition, there is also increasing evidence that ventral cortex computes action-relevant object properties and tool affordances, as well (Chen et al., 2018; Mahon et al., 2007). In this context, the ventral pathway may support object action by linking an object’s appearance with semantic knowledge about its function (Brambati et al., 2006; Buxbaum et al., 2014; Chen et al., 2018; Kalenine et al., 2011; Mahon et al., 2007) and, by virtue of connectivity to motor and premotor cortices, propagates information relevant to action planning (Brandi et al., 2014; Konen & Kastner, 2008). Consistent with these findings, temporarily disrupting tool-selective regions of the ventral pathway such as the posterior middle temporal gyrus (Vingerhoets, 2008) impairs participants’ ability to interact appropriately with tools or make accurate judgments about their use (Vingerhoets et al., 2010).

One obvious mechanism by which perception and action information in the two pathways might be reciprocally transmitted is via the extensive structural connectivity that bridges them (Baizer et al., 1991; Takemura et al., 2016; Yeatman, 2024; Yeatman et al., 2014), including that between the intraparietal sulcus (IPS) and lateral occipital cortex (LO) (Yeatman, 2024; Yeatman et al., 2014). Similar cortical connections have been documented in non-human primates, with inferior temporal regions connecting to parietal cortices (Takemura et al., 2024; Webster et al., 1994).

Unsurprisingly, given the dense structural connectivity, functional connectivity between the pathways is present both at rest (Oishi et al., 2018) and under task-evoked conditions, when participants are engaged in object recognition (Ayzenberg & Behrmann, 2022) or action-related tasks (Garcea & Mahon, 2014; Stevens et al., 2015; Walbrin & Almeida, 2021). EEG analyses of the directionality of information flow between the two pathways have revealed earlier object category decoding in dorsal than ventral regions with the former predicting the subsequent responses of the latter (Ayzenberg, Simmons, et al., 2023), perhaps an outcome of more rapid transmission of lower spatial frequency information in the dorsal magnocellular pathway (Bar et al., 2006). Furthermore, lesion and inactivation studies provide causal evidence for the directionality of these interactions, with alterations of the left anterior intraparietal and supramarginal gyrus resulting in reduced ventral temporal responses; interestingly, this is so only in response to manipulable and not to non-manipulable objects (Garcea et al., 2025; Van Dromme et al., 2016).

Although there is extensive evidence that dorsal and ventral pathways are connected and that both contribute to the processes of object recognition and action, whether this connectivity is limited to one or a few regions or is more widespread remains unclear. For example, connectivity may be limited to a subset of interactions between a single or a few specific regions in each pathway, such as those at each end of the VOF (Garcea et al., 2025). Thus, information can be transmitted from one to the other pathway to solve specific tasks, but outside of these specific cases, object recognition and action may be executed without the influence of the other pathway. Alternatively, both dorsal and ventral pathways are broadly functionally connected across the brain during object perception, and this may be so regardless of whether the task itself explicitly involves recognition or action. The implication of this latter perspective is that the processes of the dorsal and ventral pathway are deeply intertwined at almost all levels of object processing, suggesting that there is far less functional distinctiveness between the two pathways than previously believed. Adjudicating between these options will offer a clear map of the object-sensitive region as well as an expanded understanding of cortical vision.

In the current study, in two different experiments, we sought to contrast between these (and a host of other) possibilities by examining the brain-wide network properties of a single, major, functionally-defined object-selective ROI in the dorsal and in the ventral pathways. Specifically, we evaluated the extent to which the object-responsive ROI in each pathway shares a similar whole-brain connectivity structure during object viewing (even in the absence of an explicit recognition or action task). In the first experiment, we characterized the extent of the object-selective networks seeded from the dorsal and the ventral regions and then assessed the extent and strength of these overlapping connections. In the second experiment, we confirmed that the overlapping network connections are engaged for object perception more generally, rather than specifically for action-related computations, by demonstrating the same network overlap regardless of whether objects elicit action-based affordances (i.e., tools and non-tools). These widespread and distributed networks shed light on the entanglement of the neural correlates underlying object-related functions and offer a different perspective on the relationship between the dorsal and ventral visual cortical pathway.

## MATERIALS AND METHODS

### Participants

Eighteen participants (10 female, mean age = 17.6 years, SD = 7.1) completed Experiment 1, and eighteen different participants (mean age = 26.5 years, SD = 4.0) completed Experiment 2. Sample size was guided by those of previous studies investigating object processing in dorsal and ventral pathways (Bracci & Op de Beeck, 2016; Freud, Culham, et al., 2017; Jeong & Xu, 2016). These studies demonstrated that 10–15 participants provided sufficient statistical power to detect effects of interest. A subset of Experiment 1 and 2 data was analyzed and reported previously (Ayzenberg & Behrmann, 2022; Granovetter et al., 2024; Liu et al., 2019).

All 36 participants were right-handed as measured by the Edinburgh Handedness Inventory (Oldfield, 1971) with normal or corrected-to-normal vision and, for those with correction, glasses with the appropriate prescription were given to participants for use in the scanner. Participants, who were recruited from Carnegie Mellon University (CMU) community, provided informed consent under a protocol approved by the Institutional Review Board of CMU and received monetary compensation for their participation.

### Experimental design and statistical analysis

The current research sought to characterize the whole brain connectivity profile of object-responsive ROIs seeded from a selective region in the dorsal and in the ventral pathways. To this end, we calculated a range of connectivity metrics, including computing ROI-to-whole brain correlation-based functional connectivity, task-based connectivity, and effective connectivity analyses.

#### Experiment 1: Whole-brain Cortical Networks During Object Perception

**Figure 1.**
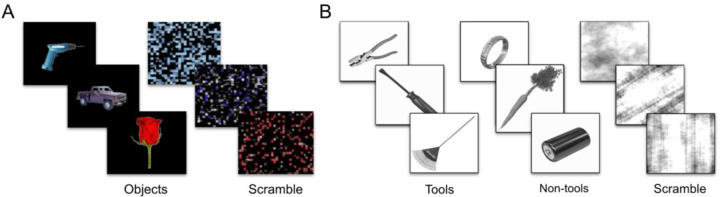
(A) Example stimuli from Experiment 1: common objects (left) and corresponding scrambled versions (right), and the objects were taken from Granovetter et al. (2024). (B) Example stimuli from Experiment 2: tools (left), non-tool objects (center), and scrambled images (right), and the stimuli were drawn from Chen et al. (2018, 2015).

In the first experiment, while in an fMRI scanner, participants viewed blocks of colored images consisting of common objects or corresponding scrambled versions of the same objects (Figure 1A). Each block of trials consisted of 16 images, each subtending 2.5° × 2° visual angle, with each image displayed centrally on the screen for 800ms with a 200ms inter-stimulus interval (16s block duration). To ensure that participants maintained attention to the stimuli, they performed an orthogonal one-back task during which they were required to press a button when an image repeated consecutively (with the repeat occurring randomly in the course of a block). Blocks were separated by 8s fixation periods. Three runs, consisting of 15 blocks each (3 blocks per condition: objects, scrambled objects, faces, houses, and words), for a total of 45 blocks, were collected per participant. For the current analyses, only the data from the object and scrambled object conditions (9 object blocks and 9 scrambled blocks) were used.

#### Experiment 2: Cortical Networks for tool versus non-tool objects

In this experiment, participants viewed blocks of images of tools, non-tool objects, or scrambled patterns presented centrally on the screen (Figure 1B). Tools were defined as objects whose physical form relates directly to function (e.g., hammer), while non-tools were objects but without direct form-function relationships (e.g., carrot), consistent with previous work (Mahon et al., 2007). Scrambled images were created by phase scrambling the tool and non-tool images. Each category (tool, non-tool objects, scrambled) was comprised of 10 unique images from Chen et al. (Chen et al., 2018; Chen et al., 2015) (Figure 1B).

Participants completed two runs (340 s total) consisting of blocks of 20 images (10 unique images per category, with each image presented twice per block). The experiment also included a fixation block (16s) that was interleaved with the other conditions, as well as 10s fixation periods at the start and end of each run. All four blocks (tools, non-tools, scramble, fixation) were presented once before repetition. Each run contained five repetitions of each condition (20 blocks in total, 5 per condition). All stimuli subtended roughly 6° of visual angle on screen, and each stimulus was presented for 700ms with 100ms ISI for a total of 16s per block. Participants performed a one-back task via button press, responding when an image repeated consecutively.

#### MRI parameters

Scanning for both experiments was conducted on a 3T MAGNETOM Siemens Prisma scanner at the CMU-Pitt BRIDGE Center (RRID:SCR_023356) using a 64-channel head coil to which a prism mirror was permitting viewing of images shown on a screen mounted on the ceiling of the bore just above the head. Participants responded using an MRI compatible glove for responding to the consecutive repeated trials. For Experiment 1, whole-brain functional images were acquired using gradient echo single-shot echoplanar imaging sequence with 69 slices, TR=2000ms, TE=30ms, flip angle=79°, voxel size=2 × 2 × 2mm^3^, FOV=212mm, using multi-band acceleration factor=3. Each run acquired 184 volumes. High-resolution T1-weighted anatomic images (TR=2300ms, TE=2.03ms, voxel size=1×1×1mm) were acquired for registration. For Experiment 2. whole-brain functional images used 48 slices, TR=1s, TE=30ms, flip angle=64°, voxel size=3×3×3mm. High-resolution T1-weighted anatomic images (TR=2300ms, TE=2.03ms, voxel size=1×1×1mm) were acquired for registration. Experiment 1 and 2 had different scanning parameters because they were originally collected as part of separate studies and no comparisons are made across these two sets of experimental data.

### Data Analyses

#### Pre-processing

Analysis included brain extraction (Smith et al., 2004) and spatial normalization to MNI 2mm space. Prior to statistical modeling, images were motion corrected, de-trended, and intensity normalized. An additional 18 motion regressors generated by FSL (Smith et al., 2004) were also included. Data were fit with a general linear model with all predictors convolved using a double-gamma hemodynamic response function. Data processing and statistical analyses were conducted using FSL (Smith et al., 2004), alongside nilearn, nibabel, and BrainIAK packages for Python (Abraham et al., 2014; Kumar et al., 2021).

#### Region of Interest (ROI) Definition

For all analyses, we used a ROI-to-whole-brain connectivity approach. Within each anatomical mask, we functionally defined an object selective ROI using object > scrambled contrast for Experiment 1, and tool + non-tool > scrambled contrast for Experiment 2. For the dorsal pathway, we functionally defined the object-selective ROI region within a posterior intraparietal sulcus (pIPS) mask created by combining IPS0 with IPS1 from the atlas created by Wang et al. (2015). For the ventral pathway, we functionally defined the object-selective region with a lateral occipital cortex (LO) mask drawn from probabilistic parcel created by Julian et al. (2012). We focused our analyses on the pIPS and LO because their importance in object processing has been extensively documented (Ayzenberg & Behrmann, 2022; Freud, Culham, et al., 2017; Grill-Spector & Malach, 2001; Malach et al., 1995). For each participant, we selected the single peak voxel from the above contrast and created a 6mm spherical ROI centered on each peak. The location of object-selective voxels were qualitatively similar across Experiments 1 and 2 despite differences in their low-level stimulus properties (such as color versus monochrome) and task parameters (see Results section).

#### Correlation-Based Connectivity Analyses

Using a ROI-to-whole-brain connectivity analysis, we mapped the functional connectivity (FC) profile of each of the dorsal and ventral pathway object-selective regions during object viewing blocks. Specifically, using a leave-one-run-out cross-validation procedure, we first identified the object-selective ROI sphere in each pathway (see ROI Definition) using two out of three runs for Experiment 1 and one run for Experiment 2. We then computed functional connectivity measures from these ROI locations using data from the held-out run. From each held-out run, we extracted the mean timeseries during the object blocks (Experiment 1: objects; Experiment 2: tool and non-tool blocks) by averaging across all voxels within each 6mm seed ROI sphere, and then computed correlations between this mean timeseries and every voxel in the brain. The resulting maps were Fisher transformed. This procedure was repeated for every possible combination of runs, and an individual’s final map was created by computing the mean across all permutations for each participant.

Group connectivity maps were created by transforming each individual map into MNI space and then averaging across all the participants. Significant voxels were determined by z-score standardizing the group map and applying FDR-correction (p < 0.05) with a proximity threshold for a cluster of five contiguous significant voxels (Ayzenberg, Granovetter, et al., 2023). This analysis examined the broad network connectivity profile of each of the dorsal and ventral ROIs in response to object viewing.

To quantify the degree of similarity between dorsal and ventral networks, we binarized each connectivity map and computed Dice coefficients, calculated as:

(1) $$DSC=\frac{2\left|\text{A}\cap \text{B}\right|}{\left|A\right|+\left|B\right|}$$

where *A ∩ B* represents the number of shared voxels and *A* and *B* represent the total number of significant voxels in the two networks being compared.

Prior to conducting repeated measures ANOVAs on Dice coefficients, values were transformed using the arcsine square root transformation (arcsin(√DSC)) to stabilize variance for proportion data bounded between 0 and 1. For between-individual comparisons, we computed pairwise Dice coefficients between each participant’s network map and all other participants’ maps, then averaged these values to yield one score per participant. For comparison, we also computed the degree of overlap for each network across participants (i.e., vs. between-participant within-network). This analysis evaluated the similarity of dorsal and ventral network profiles within an individual, relative to the consistency of each (dorsal or ventral) network map across individuals.

Next, we examined whether there were differences in the strength of the connectivity between each of dorsal and ventral ROIs and the rest of the brain. For this analysis, we parcellated the brain using a merged atlas combining the Schaefer 200-region atlas with our functionally defined Wang and Julian parcels. We replaced parcels of the Schaefer atlas that overlapped with the dorsal Wang and Julian parcels, assigning unique identifiers to pIPS (value 201) and LO (value 202). We examined the differences in connectivity strength by taking the difference of the connectivity values for each parcel in the atlas and then testing whether these differences were significantly different from 0 based on bootstrapped confidence intervals. For parcel-level classification, parcels were categorized as showing significant connectivity if they contained ≥5 voxels from the FDR-corrected group-level threshold maps.

#### Task-dependent Functional Connectivity

The next question concerned the extent to which the FC maps above were driven by visual stimulation more generally or were specifically object selective. This was especially important as the FC analysis above may simply relate to the presence of visual stimuli, regardless of whether they were objects or scrambled objects. We, therefore, quantified the overlap of dorsal and ventral networks in a task-based manner by conducting psychophysiological interaction (PPI) analyses (Friston et al., 1997). A contrastive psychological task covariate was created by assigning timepoints corresponding to object blocks a value of 1 and timepoints corresponding to scrambled blocks a value of −1 for Experiment 1. The cleaned residual timeseries from each target voxel were extracted, normalized, and concatenated across runs, then further regressed on the psychological and physiological covariates generated for those runs. A ROI-to-whole-brain functional connectivity map was generated by correlating the residual timeseries of every voxel with the interaction covariate and applying a Fisher transform and using FDR-correction (p < 0.05).

For Experiment 2, we created separate contrasts for tool > scrambled, non-tool > scrambled, and tool > non-tool, convolving the same block types with a standard hemodynamic response function. As with Experiment 1, physiological covariates were generated from each participant’s cleaned residual timeseries by extracting the timeseries from the 6-mm sphere centered on the peak voxel in each ROI. An interaction covariate was created for each participant by multiplying the psychological and physiological covariates.

This analysis examined the network profile of dorsal and ventral pathways during object perception specifically (object, tool, non-tool blocks), by contrasting them with scrambled visual stimuli, thus controlling for general visual processing. We then conducted the same overlap and connectivity strength analyses as above.

#### Partial Correlation Analysis

To characterize the unique and independent connectivity patterns of dorsal and of ventral networks while controlling for shared variance, we computed partial correlations between each pathway’s ROI timeseries and whole-brain voxels while controlling for the influence of the timeseries derived from the other pathway’s ROI. The resulting partial correlation maps were transformed to MNI space and subjected to z-score standardization followed by FDR correction (p < 0.05) with a minimum cluster size of 5 voxels. We calculated Dice coefficients between these partial correlation-based network maps using the same approach as the standard connectivity analysis.

#### Effective Connectivity

Finally, we were interested in understanding the directionality of connectivity between regions uncovered in the dorsal and ventral object networks with the rest of the brain. We, thus, used a searchlight approach to conduct Granger Causality Analyses (GCA) (Roebroeck et al., 2005; Seth et al., 2015). This analysis was implemented in each participant’s native space using Brainiak’s Searchlight class with Ball geometry (radius=2 voxels; 6 mm). For each searchlight center, bidirectional GCA was computed between the time series for each dorsal and ventral seed and the mean time series of every searchlight sphere. The time series for each searchlight sphere was extracted from the object blocks of the localizer and then standardized by z-scoring each time series across time points at TR resolution.

Following GCA computation, the resulting F-statistics were z-score standardized across all brain voxels within each participant before group analysis. Following prior work (Mijalkov et al., 2021), effective connectivity was calculated by subtracting the seed→target connectivity statistic (*F*) from the target→seed connectivity statistic using a one-time point lag (Experiment 1: 2 TR; Experiment 2: 1 TR). This difference indicates the predominant direction of information flow. Statistical significance was determined using FDR correction (p < 0.05) across all brain voxels. The final group-level searchlight maps represent the mean *F-*statistic differences across participants, indicating regions where directional connectivity with the seed ROI was consistent across the group. While fMRI’s temporal resolution limits strong inferences about the directionality of information flow, simulation studies demonstrate that GCA can resolve temporal delays of tens of milliseconds from the hemodynamic response (Deshpande et al., 2010; Katwal et al., 2013) and, thus, can provide some information about the directionality of the connectivity.

## RESULTS

### Experiment 1: A network for object processing

Our primary goal in Experiment 1 was to characterize the whole-brain network connections seeded by a dorsal and by a ventral object-selective region. We approached this using multiple complementary connectivity analyses: correlation-based FC to identify the basic network architecture, task-dependent connectivity to isolate object-specific network properties, and effective connectivity to determine the directionality of the connectivity. These analyses served to characterize the broader network of the connectivity of the dorsal and of the ventral pathway during object perception.

### Correlation-based Functional Connectivity

**Figure 2.**
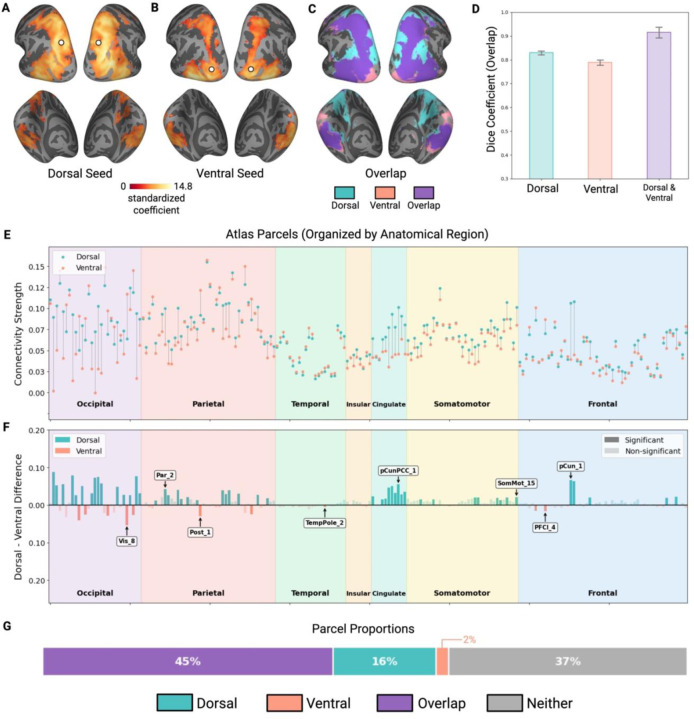
Functional connectivity during object perception. (A) Brain map showing functional connectivity from the dorsal (B) and the ventral ROIs. (A-B) White dots indicate the location of the ROI in (A) dorsal and (B) ventral pathways. (C) Overlapping (purple) and distinct (dorsal: blue; ventral: pink) regions of connectivity between the two networks. (D) Dice coefficients quantifying network overlap across three comparison types: between-subject dorsal network similarity, between-subject ventral network similarity and within-subject dorsal-ventral overlap. (E) Connectivity strength between dorsal and ventral ROI and brain parcels from the combined Schaefer-Wang-Julian atlas. Blue dots represent connectivity strength from the dorsal ROI; pink dots represent connectivity strength from the ventral ROI. (F) Magnitude differences in connectivity strength between dorsal and ventral ROIs across brain parcels. Blue bars represent regions with significantly stronger dorsal connectivity; pink bars represent regions with significantly stronger ventral connectivity. Opaque bars indicate differences that are statistically significant and transparent bars indicate differences that are not statistically significant. Labels represent selected brain regions with the largest differences between dorsal and ventral connectivity. (G) Proportion of brain parcels with significant connectivity to both pathways (purple), dorsal only (teal), ventral only (pink), or neither (gray).

We first examined the connectivity profile of object-selective ROIs in dorsal and ventral pathways in response to visual input, including objects. Both dorsal and ventral object regions showed widespread connectivity across occipitotemporal and parietal cortices (Figure 2C). More precisely, the dorsal object ROI (pIPS) showed strong connectivity to other regions throughout posterior parietal cortex, and it extended into frontal and ventral temporal regions (Figure 2B). Similarly, the ventral object ROI (LO) showed robust connectivity concentrated in occipitotemporal cortex and ventral-temporal cortex but also encompassed regions of the dorsal pathways on the lateral surface (Figure 2A). To visualize the degree of overlap between dorsal and ventral networks, we binarized the maps and overlaid them (Figure 2C), which revealed extensive spatial overlap between the two connectivity networks.

To quantify the degree of overlap seen in Figure 2C, we computed Dice coefficients between dorsal and ventral-ROI networks within the same individual and, for each network, between individuals (averaged across all pairwise comparisons per participant; see Figure 2D). Within-individual dorsal and ventral-ROI whole-brain networks exhibited 91% Dice coefficient with each other. By contrast, when just one network was compared between individuals, the dorsal network showed only 83% overlap and the ventral network 79% overlap. A repeated measures ANOVA revealed a significant main effect of overlap condition (F(2, 34) = 132.05, p < .001; post-hoc comparisons Holm-Bonferroni corrected, ps < .001). Thus, the dorsal and ventral networks underlying object perception were more similar to each other within the same participant, than each network is to itself across participants.

Although dorsal and ventral pathways exhibited a high degree of spatial overlap in the anatomical regions to which they were connected, there may, nevertheless, be quantitative differences in their network properties, namely the distribution and the strength of the connections. To examine any such differences, we analyzed the connectivity between the dorsal and ventral ROIs to every parcel defined by the merged Schaefer-Wang-Julian atlas (see Methods). The dorsal object ROI was connected to a broader set of parcels than the ventral ROI (125 vs 95 parcels), though the vast majority of connected parcels showed connectivity to both dorsal and ventral seeds (92 of 128 connected parcels; Figure 2G). The dorsal ROI also showed significantly more unique parcel connections than the ventral ROI (33 vs 3; χ^2^ = 25.00, p < .001). In terms of connectivity strength, the dorsal object ROI exhibited stronger connectivity with other dorsal parietal parcels and with frontal parcels (Figure 2E–F; blue peaks) compared to the ventral ROI. In contrast, the ventral ROI exhibited stronger connectivity than the dorsal ROI with other ventral parcels and with lateral occipital parcels (Figure 2E–F; pink peaks).

Furthermore, taking all parcels into account, there was a connectivity strength asymmetry between pathways, with 50 parcels exhibiting significantly stronger connectivity to the dorsal object ROI than to the ventral object ROI, and only 10 parcels exhibiting stronger connectivity with the ventral compared to the dorsal ROI (χ^2^ = 26.67, p < 0.001; Figure 2F). Altogether, these findings show that while dorsal and ventral pathways form highly overlapping connectivity networks during object perception, they are somewhat distinct quantitatively. Dorsal object ROI shows stronger connectivity to frontoparietal parcels, and ventral ROI shows stronger connectivity to occipitotemporal parcels. The asymmetry in both the distribution and strength of connectivity may reflect the fact that dorsal regions support diverse cognitive functions beyond object processing—including spatial attention and eye movements—that are concurrently engaged during object perception. Yet despite these differences, the vast majority of parcels showed connectivity to both dorsal and ventral seeds (Figure 2G).

### Task-dependent connectivity

Our results, thus far, reveal a high degree of spatial overlap between dorsal and ventral networks seeded from a prominent object-selective ROI during the perception of common objects, with evidence that dorsal connections are generally more widespread and stronger across cortex than ventral connections. However, it is unclear whether these connectivity patterns reflect the network underlying object perception per se, or simply the connectivity in response to any visual input more generally. To adjudicate between these possibilities, we used PPI analyses to measure connectivity when participants viewed objects and specifically contrasted this against responses to scrambled images.

**Figure 3.**
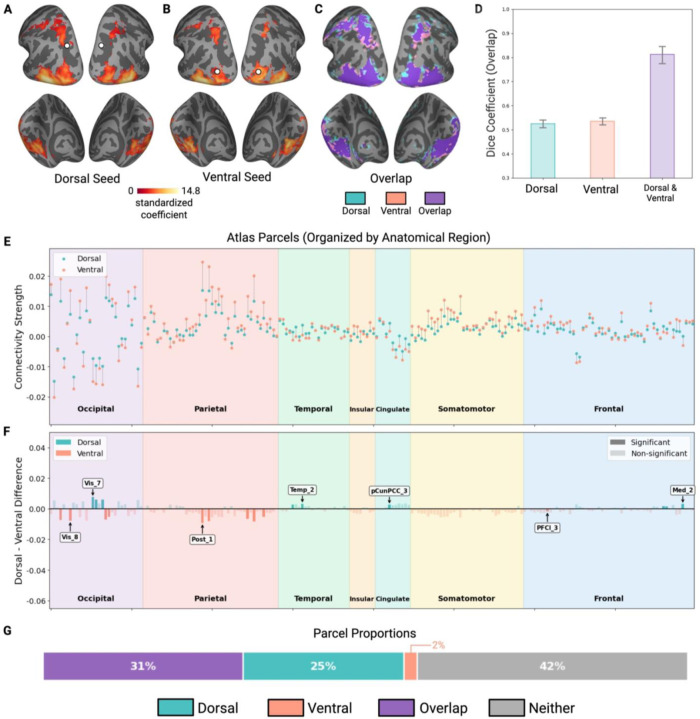
Task-dependent voxel-based connectivity during object perception. (A) Brain map showing task-dependent voxel connectivity from the dorsal ROI. (B) Brain map showing task-dependent voxel connectivity from the ventral ROI region. (C) Overlapping (purple) and distinct (dorsal: blue; ventral: pink) regions of connectivity between the two networks. (D) Dice coefficients quantifying ROI-voxel network overlap across three comparison types: within-subject dorsal-ventral overlap, between-subject dorsal network similarity, and between-subject ventral network similarity. (E) Connectivity strength between dorsal and ventral ROIs and brain parcels from the combined Schaefer-Wang-Julian atlas. Blue dots represent connectivity strength from the dorsal ROI; pink dots represent connectivity strength from the ventral ROI. (F) Magnitude differences in connectivity strength between dorsal and ventral ROIs across brain parcels. Blue bars represent regions with significantly stronger dorsal connectivity; pink bars represent regions with significantly stronger ventral connectivity. Opaque bars indicate statistically significant differences, and transparent bars indicate non-significant differences. Labels represent selected brain regions with the largest differences between dorsal and ventral connectivity. (G) Proportion of brain parcels with significant task-dependent connectivity to both pathways (purple), dorsal only (teal), ventral only (pink), or neither (gray).

Whole-brain PPI maps revealed task-dependent connectivity patterns seeded from both ROIs during object perception (Figure 3A–B). Compared to the correlation-based analysis above, both dorsal and ventral task-dependent networks were more circumscribed, with the dorsal pattern more posteriorly focused rather than spanning broad frontoparietal regions. Despite the more restricted distribution, task-dependent connectivity showed a similar profile to the correlation-based connectivity above. The dorsal object ROI (pIPS) showed task-modulated connectivity concentrated in posterior occipitotemporal and parietal regions (Figure 3B) and the ventral object ROI (LO) showed task-dependent connectivity primarily in occipitotemporal cortex and posterior parietal regions (Figure 3A).

To quantify the degree of overlap in task-modulated connectivity patterns, we computed Dice coefficients using the same approach as the functional connectivity analysis above. Within-individual, the dorsal and ventral task-dependent whole-brain networks exhibited 81% Dice coefficient. By contrast, when each network is compared across individuals, both dorsal and ventral networks showed lower overlap (52.5% and 53.5%, respectively) than the within-individual correlation. A repeated measures ANOVA on these three conditions revealed a significant main effect of overlap condition (F(2, 34) = 253.96, p < .001), with post-hoc comparisons showing that dorsal-ventral overlap within-individual was significantly higher than either dorsal (p < .001) and ventral (p < .001) overlap across participants (Figure 3D). This task-based pattern mirrors the correlation-based functional connectivity results above, suggesting that whole-brain object-specific connectivity patterns for the two pathways are highly similar both within- and across-individuals but to a greater extent in the former than latter.

To examine quantitative differences in the task-dependent network of the two pathways, we analyzed connections from dorsal and ventral ROIs to each parcel defined by the merged Schaefer-Wang-Julian atlas (see Methods). Although the majority of connected parcels showed connectivity to both pathways (65 of 118 significantly connected parcels), the dorsal ROI was connected to more parcels than the ventral ROI (115 vs 68), with significantly more unique dorsal than ventral parcel connections (50 vs 3; χ^2^ = 41.68, p < .001; Figure 3G). In terms of connectivity strength, unlike the correlation-based analysis, in which the dorsal ROI showed stronger connectivity than the ventral ROI to the majority of atlas parcels, we found no significant asymmetry in task-dependent connectivity strength, with 9 parcels exhibiting significantly stronger connectivity with the dorsal object ROI compared to the ventral object ROI, and 10 parcels showing the reverse (χ^2^ = 0.05, p = 0.82; Figure 3F).

### Partial Correlation Analysis

As is apparent from prior analyses, there is substantial overlap between ventral and dorsal whole-brain connectivity networks during object perception. While we have suggested that these networks are independent and yet overlap, an alternative explanation for this outcome is that the observed overlap is the result of information from one pathway propagating through the other—for example, ventral information reaching frontal cortex via the dorsal ROI. In this view, one pathway has no independent connection to a given region, and the apparent connectivity arises from the shared variance between the dorsal and ventral ROIs, such that, the correlation between the ventral ROI and frontal cortex may be a byproduct of its correlation with dorsal. To address these concerns, we conducted partial correlation analyses controlling for the shared variance between pathways. Specifically, we computed dorsal ROI connectivity while controlling for the ventral ROI timeseries, and ventral ROI connectivity while controlling for the dorsal ROI timeseries.

**Figure 4:**
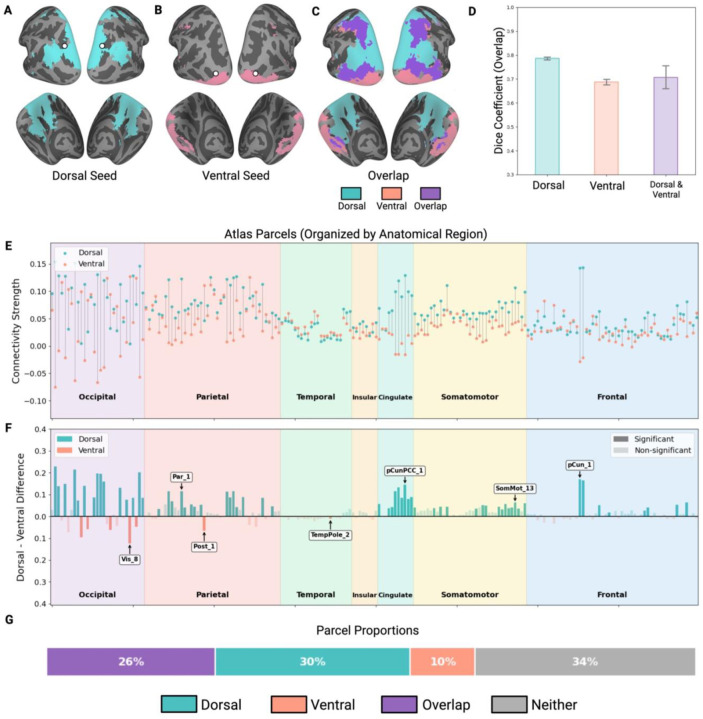
Pathway-specific connectivity after partial correlation analysis. (A) Brain map showing dorsal-specific regions (dorsal partial correlation map with overlapping regions subtracted for visualization clarity). (B) Brain map showing ventral-specific regions (ventral partial correlation map with overlapping regions subtracted for visualization clarity). (C) Composite map showing all three categories: dorsal-specific (blue), ventral-specific (pink), and regions with significant connectivity to both pathways (purple). (D) Dice coefficients quantifying network overlap across three comparison types: within-subject dorsal-ventral overlap after partial correlation, between-subject dorsal network consistency, and between-subject ventral network consistency. Error bars represent 95% confidence intervals. (E) Connectivity strength for regions with overlapping connectivity (purple) compared across dorsal and ventral ROIs, organized by brain parcel from the combined Schaefer-Wang-Julian atlas. (F) Magnitude differences in connectivity strength between dorsal and ventral ROIs across brain parcels. Blue bars represent regions with significantly stronger dorsal connectivity; pink bars represent regions with significantly stronger ventral connectivity. Opaque bars indicate statistically significant differences and transparent bars indicate non-significant differences. Labels represent selected brain regions with the largest differences between dorsal and ventral connectivity. (G) Proportion of brain parcels with significant independent connectivity to both pathways (purple), dorsal only (teal), ventral only (pink), or neither (gray).

After controlling for shared variance, each pathway showed distinct but also overlapping connectivity (Figure 4C). More precisely, the dorsal object ROI (pIPS) showed strong connectivity to areas in occipital and parietal cortex, as well as frontal and ventral temporal regions (Figure 4A). In contrast, the ventral object ROI (LO) showed robust connectivity concentrated in occipital cortex primarily in earlier and more inferior regions of visual cortex (Figure 4B). To visualize the degree of overlap between dorsal and ventral networks, we binarized the maps and overlaid them (Figure 4C), which revealed extensive spatial overlap between the two connectivity networks. To quantify the degree of overlap visible in Figure 4C, we computed Dice coefficients between dorsal and ventral-ROI networks within the same individual and then, for each network, between individuals (see Figure 4D).

Within-individual, dorsal and ventral-ROI whole-brain networks exhibited 70.6% overlap with each other (95% CI [65.2%, 76.1%]). By contrast, when just one network was compared between individuals, the dorsal network showed 78.6% consistency (95% CI [77.3%, 80.0%]) and the ventral network 68.7% consistency (95% CI [66.3%, 71.2%]). Levene’s test on the transformed data indicated significant heteroscedasticity across comparison types (F(2, 51) = 11.65, p < .001), so we used Welch’s ANOVA, which revealed a significant main effect of comparison type (F(2, 51) = 30.61, p < .001). Post-hoc tests with Holm-Bonferroni correction showed that between-subject dorsal consistency was significantly higher than both within-subject overlap (p = .016) and between-subject ventral consistency (p < .001). Within-subject overlap and between-subject ventral consistency did not differ significantly (p = .42).

Notably, this pattern differs from the analyses above, in which within-subject overlap was highest. Here, the dorsal pathway maintained high connectivity consistency (79.1%) even after controlling for the ventral signal, whereas ventral consistency dropped substantially (from 79% to 69.5%). This suggests that the ventral pathway’s connectivity to other brain areas may be partly explained by its shared variance with the dorsal pathway, whereas dorsal connectivity is more independent.

Although dorsal and ventral pathways exhibited overlap in the regions to which they were connected even after controlling for shared variance, there may, nevertheless, be quantitative differences in their network properties. To examine these differences, we analyzed connectivity patterns using the merged Schaefer-Wang-Julian atlas (see Methods). Of 200 parcels, 55 showed significant connectivity to both pathways even after controlling for shared variance (Figure 4C), spanning frontal, somatomotor, parietal, occipital, and temporal cortices. The dorsal pathway showed significant unique connectivity with 61 parcels, concentrated in visual cortex, posterior parietal cortex, precuneus, posterior cingulate, somatomotor cortex, and frontal eye fields (Figure 4A), while the ventral pathway showed significant unique connectivity with 19 parcels, concentrated in early visual cortex and temporal pole (Figure 4B). This asymmetry (χ^2^ = 22.05, p < .001) indicates greater independent dorsal connectivity. In terms of connectivity strength within the 55 overlapping parcels, the dorsal ROI exhibited significantly stronger connectivity than the ventral ROI in 11 parcels, while the ventral ROI showed stronger connectivity in 4 parcels, with no significant asymmetry in strength (χ^2^(1) = 3.27, p = .071; Figure 4E–F). These findings demonstrate that dorsal and ventral pathways maintain both shared and pathway-specific connectivity even when accounting for the correlation between the ROIs, with dorsal regions showing more independent parcel connections. Within the overlapping parcels, however, connectivity strength was comparable across pathways.

### Effective connectivity

Thus far, we have demonstrated that both general and task-specific connectivity patterns are highly overlapping for dorsal and ventral regions. However, neither of these findings address the directional nature of connectivity between dorsal and ventral pathways and the rest of the brain. Based on previous EEG evidence showing earlier object category decoding in dorsal compared to ventral regions, we hypothesized that dorsal regions would show a directional influence on ventral processing rather than vice versa (Ayzenberg, Simmons, et al., 2023; Bar et al., 2006). We examined the directionality of object information from dorsal and ventral pathways to the rest of cortex using object-selective data and GCA with a whole-brain searchlight approach.

**Figure 5.**
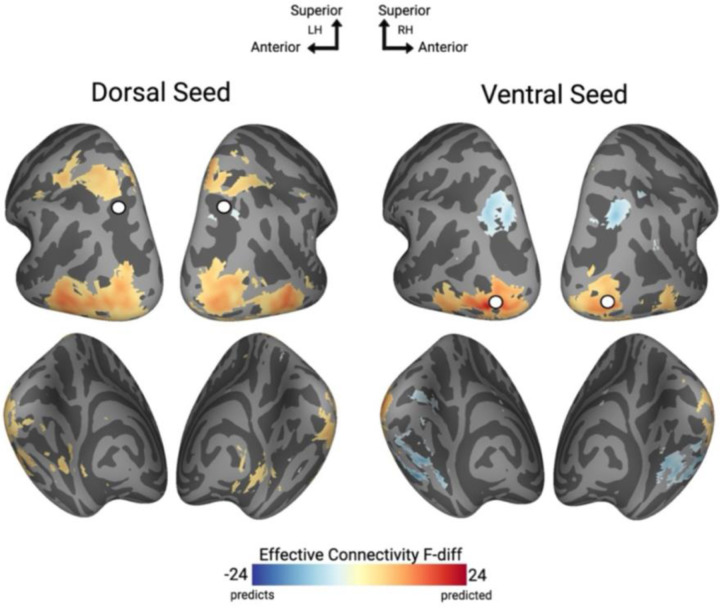
A) Effective connectivity group map, seeds are demarcated with white spheres. Blue regions predict the seed and red regions are predicted by seed.

Whole-brain searchlight analysis revealed predominantly a dorsal-to-ventral directional connectivity pattern during object perception (Figure 5A–B). Specifically, the object-selective dorsal object seed predicted anterior regions of parietal cortex as well as lateral portions of the ventral pathway (Figure 5A) and was predicted by posterior visual regions (Figure 5A blue). Similarly, the ventral object seed predicted more anterior portions of the lateral ventral pathway (Figure 5B red) and was predicted by posterior parietal cortex and medial portions of the ventral pathway (Figure 5B blue). Thus, although fMRI cannot provide strong evidence for the directionality of information flow, consistent with our prediction, these findings provide evidence consistent with existing findings that dorsal regions may exert a directional influence on ventral processing during object perception.

### Experiment 2: Tools and non-tools processing

Experiment 1 revealed that dorsal and ventral visual pathways show largely overlapping patterns of connectivity during the viewing of objects, though the dorsal pathway was consistently connected to a broader set of regions and, in the correlation-based analysis, showed stronger connectivity than the ventral pathway. Because the object images included in Experiment 1 contained both tool and non-tool images, it is possible that the stronger dorsal connectivity and the degree of overlap observed in Experiment 1 may be explained by the presence of tool images, rather than by objects more generally. Indeed, tool images are assumed to be a special class of stimuli that disproportionately engage dorsal regions because of their strong action affordances (Chen et al., 2018; Mahon et al., 2007), potentially accounting for the stronger dorsal connectivity observed in Experiment 1. This raises the question of whether the overlapping connectivity patterns from Experiment 1 reflect general object processing or are driven specifically by tool stimuli. To address this, Experiment 2 examined the similarity between dorsal and ventral whole-brain connections separately for tools and non-tool stimuli. We predicted that if the findings from Experiment 1 were driven just by the viewing of tools, we would observe overlapping dorsal and ventral network connectivity only when tools were viewed, but not when non-tool objects were viewed.

### Correlation-Based Functional Connectivity

**Figure 6.**
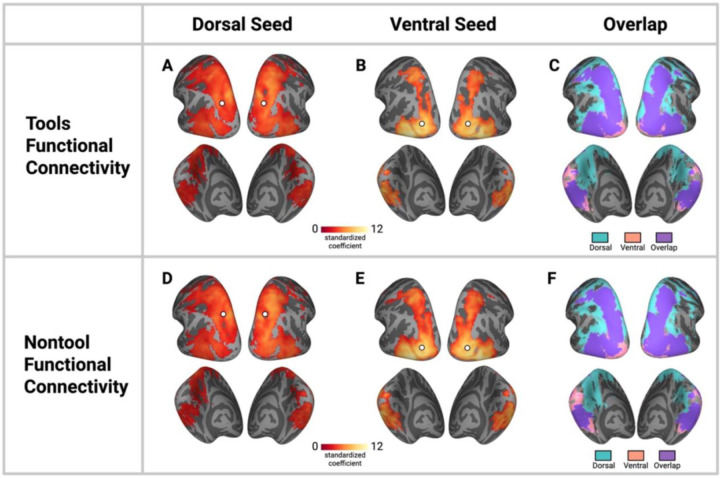
Functional connectivity patterns for (A-C) tools and (D-F) non-tools. Dorsal ROI (pIPS) connectivity for tools (A) and non-tools (D). Ventral ROI (LO) connectivity for tools (B) and non-tools (E) Color bar represents standardized coefficients. Overlap maps showing regions of shared connectivity between dorsal and ventral networks for tools (C) and non-tools (F). Purple regions in overlap maps indicate overlapping connectivity between the two pathways.

Both tools and non-tools stimuli demonstrated functional connectivity patterns similar to those observed in Experiment 1. There was significant ROI-to-whole-brain connectivity correlations for each of the dorsal and ventral ROIs for both tool and non-tool categories (Figure 6A–D). Both dorsal and ventral object ROIs showed widespread connectivity across occipitotemporal, parietal, and frontal cortices for both tools and non-tools. For both tool and non-tool categories, the dorsal object ROI (pIPS) showed strong connectivity in its characteristic regions—posterior parietal and frontal cortex —but also extended into ventral temporal regions, and the ventral object ROI (LO) showed connectivity concentrated in occipitotemporal and ventral-temporal cortex, but also encompassed dorsal pathway regions on the lateral surface. To visualize the degree of overlap between dorsal and ventral networks for each object category, we binarized the maps and overlaid them (Figure 6E–F), and this revealed extensive spatial overlap between the two networks regardless of object category.

We quantified the degree of overlap for each object category by computing Dice coefficients between dorsal and ventral-ROI networks within individual and then between individuals. For both tools and non-tools, within-individual dorsal and ventral-ROI networks exhibited approximately 92.3% and 91.9% dice coefficients respectively. By contrast, when compared between individuals, the dorsal network showed 83.0% overlap and the ventral network approximately 78.9% dice coefficient with their respective counterparts. A 2×3 repeated measures ANOVA with factors Object Category (tools, non-tools) and Comparison Type (within-subject, between-dorsal, between-ventral) quantified these differences with a significant main effect of Comparison Type (F(2, 34) = 225.52, p < .001), with post-hoc comparisons (Holm-Bonferroni corrected) showing that dorsal-ventral overlap within-individual was significantly higher than either dorsal and ventral overlap across participants (ps < .001). Critically, there was no main effect of Object Category (F(1, 17) = 0.08, p = .778) and no interaction (F(2, 34) = 0.40, p = .674), indicating that this pattern holds equally for both tools and non-tools. Thus, consistent with Experiment 1, the dorsal and ventral networks underlying object perception are more similar to each other within the same participant than each network is to itself across participants, and this pattern reflects general object processing rather than tool-specific connectivity.

Although dorsal and ventral ROIs exhibited a high degree of overlap in the regions to which they were connected (above), there are nevertheless differences in the magnitude of their connectivity to different regions. To examine these connectivity strength differences, we analyzed the connections between dorsal and ventral ROIs to each parcel defined by the merged Schaefer-Wang-Julian atlas (see Methods). For tools, dorsal object ROIs exhibited stronger connectivity with 81 regions while ventral exhibited stronger connectivity with only 11 regions (χ^2^ = 53.26, p < .001). Non-tools showed a similar asymmetry, with dorsal ROIs exhibiting stronger connectivity with 107 regions compared to 9 regions for ventral ROIs (χ^2^ = 82.79, p < .001). The proportion of dorsal-dominant parcels did not differ significantly between tools and non-tools (χ^2^(1) = 1.04, p = .31). Thus, consistent with the correlation-based analyses of Experiment 1, the dorsal pathway showed overall stronger connectivity magnitude to a greater number of regions than the ventral pathway, regardless of whether participants were viewing tools or non-tools. Altogether, these findings demonstrate that both tools and non-tools engage highly similar dorsal and ventral networks with nearly identical within-participant overlap patterns (92% for both categories). Furthermore, as in Experiment 1, we observed greater connectivity weight asymmetry favoring dorsal regions.

### Direct comparison of tool and non-tool networks

**Figure 7.**
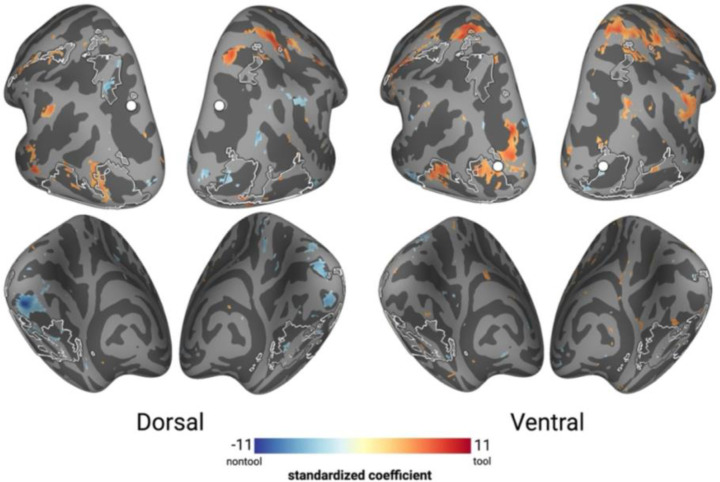
Task-based connectivity for tools over non-tools. Red regions indicate stronger connectivity for tools; blue regions indicate stronger connectivity for non-tools. Seeds are marked in white, Black/white outlines show overlapping network regions from Experiment 1’s task-dependent connectivity analysis. Color bar represents standardized coefficients (p < 0.05, FDR-corrected).

In the final analysis, we directly compared tool and non-tool networks by contrasting these conditions using PPI. These analyses revealed few differences between tools and non-tools, regardless of whether dorsal or ventral seed regions were used (Figure 7). The differences that did emerge appeared largely outside the overlapping regions identified in Experiment 1 (Figure 7, white outlines). Specifically, tools showed greater connectivity in more anterior parietal regions compared to non-tools, consistent with the possibility of tool-specific affordance processing and engagement of motor cortices (Chen et al., 2018; Mahon et al., 2007).

The goal of Experiment 2 was to determine whether the findings from Experiment 1 could be accounted for merely by the presence of tools in the stimulus set. Here, we showed that tools and non-tools showed similar connectivity patterns to each other and with the overlapping network regions reported in Experiment 1. As such, the interpretation of the Experiment 1 data and documented connectivity profiles indicates that object perception per se (rather than tool perception) engages overlapping network regions, and that the extensive dorsal-ventral overlap observed in Experiment 1 reflects object processing per se rather than tool-specific representations.

## DISCUSSION

The goal of the current study was to characterize the nature, extent and overlap of object-selective connectivity between dorsal and ventral visual regions. While many previous studies have already documented object-selective responses in both parietal and ventral cortex (Freud et al., 2016; Jeong & Xu, 2016; Konen & Kastner, 2008), the breadth of the object network distribution from each pathway has not been evaluated. In two separate experiments and using three connectivity metrics (functional, task based and directional), our results revealed a remarkable degree of overlap in connectivity between the two pathways generated across the entire brain from a dorsal and a ventral seed while participants completed a task-free object viewing paradigm. Indeed, the similarity of the dorsal and ventral object network distributions within an individual was consistently greater than the similarity of each network across individuals, revealing superior intra- than inter-subject homogeneity as one might expect from a well-connected network. Importantly, this overlap persisted when we controlled for any shared variance between pathways and for the visual input, with similar connectivity maps for both action-relevant (tools) and non-action relevant (non-tools) objects. That the network connectivity was specific in response to objects (i.e., modulated by object viewing rather than any visual input such as scrambled images) attests further to the specificity of the overlap of the two networks. Last, using a Granger causality approach, we confirmed previous findings that dorsal object-selective regions predicted ventral object-selective regions rather than vice versa. Together these findings suggest that the interactions between the dorsal and ventral pathways are not limited to just the well-known pIPS and LO object regions (Freud et al., 2015; Walbrin & Almeida, 2021) nor to the established connectivity between the superior parietal lobule and fusiform gyrus (Garcea & Mahon, 2014; Garcea et al., 2025), but that each of these regions is deeply interconnected with whole-brain networks that overlap substantially.

### Object Specific Connectivity

Three alternative explanations for the extensive overlap we documented should be considered. The first possibility is that the correlation-based overlap we observed in Experiment 1 resulted merely from activation in response to any visual input and, thus, does not bear on object-based networks specifically. We evaluated this possibility using a task-based connectivity analysis (PPI) to regress out activity related to the scrambled object condition, a proxy for non-object visual input, and still found substantial overlap between dorsal and ventral networks when only objects remained in the stimulus set. However, a second possibility is that these connectivity patterns emerged because some of the objects we displayed had affordances for action, thereby requiring both dorsal and ventral processing and perhaps to a greater extent from dorsal cortex, which we also observed.

To determine the merit of this account, we examined the connectivity networks of dorsal and ventral object networks separately for images of tools, which have clear action affordances, and for non-tools which do not. Our results showed that both tools and non-tools elicited similar connectivity overlap patterns, demonstrating that our findings of dorsal and ventral regional overlap reflected the processing of object properties per se rather than tool-specific affordance. A final possibility is that the overlap we observed does not reflect distinct contributions of dorsal and ventral pathways but is the result of the collinearity among dorsal and ventral seed regions which arises as a function of the inherent structural connections such as the VOF (Yeatman et al., 2014). In this view, the presence of brain-wide overlapping connections is the result of information from one pathway being propagated through the second pathway, rather than a direct connection. If the overlap reflects collinearity rather than independent connections, then removing the shared variance between pathways should reduce the overlapping connectivity patterns. We tested this possibility by computing connectivity maps for each seed region after regressing out the signal of the other region, thereby isolating the functional correlations of each pathway. Although this analysis reduced the amount of overlap between pathways, there nevertheless remained a high degree of overlap. Specifically, dorsal-ventral overlap within an individual was comparable to the overlap of the ventral network across individuals. However, this analysis also revealed that the dorsal pathway has a high degree of connectivity that is independent of the ventral pathway. These findings suggest that dorsal and ventral object regions show overlap in their whole-brain connectivity networks that cannot be fully explained by any type of visual input, object affordances, or the collinearity among regions, though the ventral pathway’s connectivity may be more dependent on its shared variance with the dorsal pathway than vice versa.

### Extensive Spatial Overlap Between Dorsal and Ventral Pathway Connectivity

Although it is accepted that dorsal and ventral pathways communicate with each other, the extent to which they also share connections across the brain has not been investigated. By examining seed-to-whole brain functional connectivity from both dorsal and ventral object seeds, rather than just connectivity between the two seed regions, our approach revealed that the connectivity with each pathway is shared across a broader network of regions. Moreover, the persistence of high overlap even for both tool and non-tool objects indicates this shared connectivity reflects the basic principle of object processing, not one specific to objects with action affordances.

This organization might reflect the demands of natural behavior, where recognition, spatial perception, and action planning often occur in parallel (Gibson, 1979; Hayhoe & Ballard, 2005). One possible account draws on the ecological approach to visual perception, where object perception automatically includes the extraction of affordance values (Borghi & Riggio, 2015; Gibson, 1979), meaning that viewing objects inherently activates both identity and action-related information. However, given that we observed equivalent overlap for both tools and non-tools, a more likely account is that this organization reflects the specific demands of object recognition rather than affordance processing per se. Such organization may arise because object perception inherently involves the simultaneous representation of both spatial properties, such as an object’s location, and featural properties such as its shape (Cox et al., 2005; Golomb et al., 2014), which are computed by dorsal and ventral pathways respectively.

One possibility is that the pathways exert reciprocal constraints on each other, with shape and spatial dorsal variables modulating responses in ventral regions even for objects that are merely viewed (Almeida et al., 2014). A more parsimonious possibility is that similar object representations are simply available in both pathways; indeed, both pathways show a similar transition from shape to category representation along the posterior-to-anterior anatomical axis (Bracci & Op de Beeck, 2016). In our data, dorsal and ventral connectivity networks still overlap when the signal from the other pathway is controlled for and connectivity is generated from pathway-unique correlations.

These findings further challenge strong views of dorsal and ventral pathways as largely independent networks for object recognition and action, and go beyond claims that they primarily interact with each other via one node or a small number of nodes. Instead, our results show that ventral and dorsal object networks are deeply intertwined across the brain, suggesting object perception inherently involves the coordinated activation of both pathways. This finding is consistent with more recent cortical organization accounts of large-scale functional gradients with integrative regions without full overlap of all areas (Blauch et al., 2026; Huntenburg et al., 2018; Margulies et al., 2016).

### Pathway Specialization and Connectivity Patterns

While our findings demonstrate extensive overlap between dorsal and ventral object networks, the results also support some independence of the two pathways. Specifically, after controlling for the indirect connections of the other pathway, we found parcels in frontal and anterior parietal cortex, likely involved in action-related processes, which were uniquely connected to dorsal pathway. By contrast a small number of parcels in posterior occipitotemporal cortex, which are likely involved in detailed visual form analysis, were uniquely connected to the ventral pathway.

In addition to spatial differences, we also observed differences in connectivity strength patterns within overlapping regions. Specifically, across most of our analyses we found that dorsal object regions exhibited stronger connectivity to other cortical areas than ventral object regions. In fact, the effective connectivity results suggested that, under general conditions, dorsal regions may serve as a source of the input to the majority of overlapping regions. The broader connectivity of dorsal regions during general visual processing also aligns with evidence that dorsal parietal cortex contributes to diverse cognitive functions beyond object processing, including number perception, abstract reasoning, and structure learning (Acuna et al., 2002; Bueti & Walsh, 2009). Thus, despite extensive overlap in their connectivity profiles, there are nevertheless functions that involve primarily just the dorsal or just the ventral with dorsal-specific connectivity concentrated in anterior parietal and motor regions and ventral-specific connectivity concentrated in posterior occipitotemporal regions.

### Reconciling Overlap With Independent Functions

Overlapping connectivity networks do not imply an obvious replication of function. In addition to differences in the breadth of connectivity, there were also differences in the strength of network connectivity from the dorsal or ventral object ROIs. Moreover, dorsal and ventral object ROIs showed similar network connections even after regressing out the signal of the other network, suggesting that each pathway contributes unique information to the brain-wide object perception network. Finally, we also found parcels that were exclusively connected with one pathway, such as the connections of motor cortex with the dorsal object seed. These findings are consistent with extensive evidence from patients that shows that the dorsal pathway contributes to object processes, like 3D shape even when there is extensive ventral pathway damage (Freud, Ganel, et al., 2017). We propose that some independence remains even within this highly overlapping dorsal-ventral network.

Moreover, data from lesion and inactivation studies further support the idea that the dorsal pathway has a modulatory role on the ventral pathway functions, rather than the other way around. For instance, lesions to anterior intraparietal sulcus and supramarginal gyrus specifically reduce fusiform responses to manipulable objects while sparing responses to other categories, providing causal evidence for parietal influence on ventral object processing (Garcea et al., 2025), while other studies in macaques demonstrate that temporary parietal inactivation impacts ventral responses more strongly than the reverse direction (Van Dromme et al., 2016). Together, this convergence of electrophysiological and causal evidence with our fMRI findings strengthens confidence in the observed directional pattern.

### Future directions

The entire focus of this research has been on connectivity in response to photographs. We know, however, that 3D or real world objects elicit different responses than images in perception (Fairchild et al., 2021; Marini et al., 2019), recognition (Holler et al., 2019) and attention (Gomez et al., 2017). The 2D versus real world (naturalistic) activation profiles are not just quantitative but are also qualitatively different (Holler et al., 2020) (see (Snow & Culham, 2021)). Whether similar network overlap profiles are also elicited by real world objects remains to be determined in future studies.

A further focus for future work concerns the causal interactivity between dorsal and ventral cortex. Here, and in previous work (Ayzenberg, Simmons, et al., 2023), we have shown that dorsal cortex precedes and predicts activity in ventral cortex but in neither case does this involve a direct manipulation. Transient inactivation using Transcranial Magnetic Stimulation (TMS) or Transcranial Direct Current Stimulation (TDCS) might permit more causal relationships between the activation of networks from each of the two pathways. Last, investigations of patients with circumscribed damage to one or the other pathway continues to offer promising ways of understanding the necessity and sufficiency not just of two pathways but of their distributed connectivity, as well.

### Conclusions

The distinction between a dorsal pathway for spatial relations and action processing, and a ventral pathway for visual recognition is a fundamental organizing principle of visual neuroscience. However, research has increasingly shown widespread connectivity between specific regions of interest in each pathway, such as pIPS and LO. In the current study, we found that such connectivity is not limited to connections between these major regions of interest in each pathway but that the functional network is distributed widely across many regions of the brain. We also documented extensive overlap between the single-pathway networks even in the absence of an explicit action or object recognition task. Ultimately, the processes of space, action, and recognition may be inherently entangled with one another and automatically engaged and activated in response to the viewing of visually presented objects.
